# Semantic alignment of the German Human Genome-Phenome Archive metadata model in Europe’s genomics field

**DOI:** 10.1038/s41597-026-06575-y

**Published:** 2026-02-11

**Authors:** Karoline Mauer, Anandhi Iyappan, Simon Parker, Bilge Sürün, Galina Tremper, Paul Menges, Léon Kuchenbecker, Koray Kirli, Joachim L. Schultze, Sven Nahnsen, Thomas Ulas

**Affiliations:** 1 Systems Medicine, German Center for Neurodegenerative Diseases (DZNE) e.V, Bonn, Germany; 2 PRECISE Platform for Single Cell Genomics and Epigenomics, German Center for Neurodegenerative Diseases (DZNE), University of Bonn and West German Genome Center, Bonn, Germany; 3 Structural and Computational Biology Unit, European Molecular Laboratory (EMBL), Heidelberg, Germany; 4 German Human Genome-Phenome Archive (GHGA, W620), German Cancer Research Center (DKFZ), Heidelberg, Germany; 5 Juristische Fakultät, Universität Heidelberg, Heidelberg, Germany; 6 Applied Bioinformatics, Department of Computer Science, University of Tübingen, Tübingen, Germany; 7 Federated Information Systems, German Cancer Research Center (DKFZ), Heidelberg, Germany; 8 Complex Medical Informatics, Medical Faculty Mannheim, Heidelberg University, Mannheim, Germany; 9 Core Facility Omics IT and Data Management (ODCF, W610), German Cancer Research Center (DKFZ), Heidelberg, Germany; 10 Genomics and Immunoregulation, Life & Medical Sciences (LIMES) Institute, University of Bonn, Bonn, Germany; 11 Cluster of Excellence ImmunoSensation2 (EXC 2151) ‘ImmunoSensation2 - the immune sensory system’, University of Bonn, Bonn, Germany; 12 Quantitative Biology Center (QBiC), University of Tübingen, Tübingen, Germany; 13 Department of Computer Science, Eberhard-Karls University of Tübingen, Tübingen, Germany; 14 M3 Research Center, University Hospital, Tübingen, Germany; 15 Cluster of Excellence iFIT (EXC 2180) ‘Image-Guided and Functionally Instructed Tumor Therapies’, Eberhard-Karls University of Tübingen, Tübingen, Germany; 16 Institute for Bioinformatics and Medical Informatics (IBMI), Eberhard-Karls University of Tübingen, Tübingen, Germany

**Keywords:** Research management, Genetic databases

## Abstract

Legal and technical developments drive data sharing via federated infrastructures, especially in the field of human omics. This requires interoperability across technical, syntactic, organizational, and semantic layers. The German Human Genome-Phenome Archive (GHGA) has been building a national, federated infrastructure for secure sharing of human omics data. As part of its mission to enhance interoperability and to promote reliable data sharing, a detailed crosswalk analysis was conducted comparing the GHGA metadata model with four other domain-relevant standards and metadata models: EGA (Submission API and model draft), FAIR Genomes and ISA-tab. The analysis aimed at identifying semantic consensus fields to define datasets in the context of human omics by forward mapping (GHGA model to external models). Backward mapping (external models to GHGA) focused on spotting gaps in GHGA’s semantic metadata representation. Forward mapping showed overall similar property coverage across models, aligning with MINSEQE. Backward mapping showed greater model heterogeneity. None of the identified information gaps spanned across all models. These findings highlight the detail and adaptability of the GHGA metadata model.

## Introduction

Genomic research has made remarkable strides in recent years, driven by advancements in high-throughput sequencing, artificial intelligence (AI), and integrative multi-omics approaches. These innovations have expanded the understanding of genetic diseases, enabled precision medicine, and facilitated the development of targeted therapies. Whole-genome sequencing (WGS) and transcriptomics, coupled with AI-driven analytics, have significantly improved diagnostic accuracy and treatment personalization, making genomic medicine a cornerstone of modern healthcare^1^. These developments have transformed biomedicine into a data intensive discipline. Vast, heterogeneous datasets now drive discovery and clinical decision-making, but they also pose substantial challenges in data management and interpretation. Large datasets can only be put into meaningful context when shared beyond institutional and national borders. These requirements have lately given rise to a global focus on FAIR research data^2^. However, these advances have also exposed a critical challenge, many existing metadata infrastructures are not equipped to handle the scale, complexity, and privacy requirements of modern omics data.

Metadata, i.e., data about data, is crucial to meaningfully describe data and to provide necessary context. The differentiation between data and metadata is context-specific and varies with the use case. The definition of the term “metadata” itself is also ambiguous and used inconsistently, adding another layer of non-uniformity that needs a solution in order to achieve interoperability^3^. In the realm of biomedical omics, “data” is often considered to be the output of a sequencing experiment and the subsequent downstream analysis, and the “metadata” is the context in which the data were created, such as origin, experimental protocols, or even clinical data describing the case. Metadata can also cover administrative information explaining consent information and access conditions^4^.

Robust and interoperable metadata frameworks are essential to harness the full potential of real-world omics data in personalized medicine. A major challenge lies in the rapid technological advances that require continuous adaptation of metadata models (e.g., for new sequencing methods) and in the fragmented landscape of existing standards that capture only partial metadata (e.g., only clinical data or only experimental metadata). While multiple standards with defined classes and attributes exist for clinical data (e.g. OMOP CDM (Observational Medical Outcomes Partnership Common Data Model^5^) and HL7 FHIR (Fast Healthcare Interoperability Resource)^6,7^), or for single experimental approaches (e.g. minSCe for single-cell RNAseq experiments^8^) there is no overarching metadata standardization for genomics experiments that goes beyond minimum information recommendations (e.g. MINSEQE^9^). Further guidelines, such as the GA4GH Experiments Metadata Standard, are currently under development (https://www.ga4gh.org/product/experiments-metadata-standard/). Traditional metadata schemas, often tailored to a certain experimental approach or use case, therefore struggle with interoperability, standardization, and scalability, limiting their ability to support emerging technologies. This includes machine learning-driven genomic analysis and federated data sharing^10,11^ or collecting data from multiple repositories, which further reinforces the fragmentation of data into isolated silos.

In the European landscape, alignment with ethical and legal principles is central to enabling responsible data exchange. Metadata models for human omics data often fall short due to differences in use cases, metadata model scopes, and inconsistent implementation of opening and/or closing clauses of the General Data Protection Regulation (GDPR), leading to additional challenges in cross-border data exchange^12^. Researchers have expressed concerns that the GDPR’s emphasis on individual consent and data protection mechanisms discourages institutions from participating in open data-sharing initiatives. For example, in pediatric cancer research, scientists have found that GDPR restrictions limit access to vital datasets, hampering research^13^. The reluctance to share data due to legal and ethical concerns further exacerbates the challenge, particularly when studying rare diseases that require extensive international collaboration. The lack of a unified interpretation of GDPR across EU member states leads to inconsistencies in data governance, making it difficult to establish large-scale collaborative projects in genomics^14^. At the same time, GDPR remains essential for protecting individual privacy and is thus an essential foundation for safe, legally sound, and trustworthy data infrastructures^15^.

The absence of standardized metadata models across European countries also hampers interoperability in large-scale European initiatives. Despite efforts such as the *1 + Million Genomes* (1 + MG) Initiative^16^, which aims to create a federated infrastructure for secure genomic data sharing, different national regulations and data governance frameworks continue to hinder seamless integration across borders. The European Genome-phenome Archive (EGA)^17^ and the European Genomic Data Infrastructure (GDI) have made efforts to establish federated, interoperable solutions, but differences in governance models, metadata frameworks, and data access regulations still create obstacles. Another challenge is the emerging trend towards data-driven personalized medicine and secondary use data from routine health care, often referred to as “real-world data”, in addition to controlled study cohort data^18^. These data structures vary significantly in their granularity and standardization measures, both of which tend to be higher for cohort data than for real-world data. Despite being less standardized and harder to access, real-world data, and especially real-world omics data, are essential for robust data analysis, real-world evidence and to drive personalized medicine^19^. For data portals in the health sector and their metadata models, it is imperative to be able to incorporate data from clinical studies, as well as real-world data^18^.

The German Human Genome-Phenome Archive (GHGA) aims to tackle the shortcomings of existing metadata issues such as lack of semantic interoperability and adaptability by building a federated, secure, and standardized infrastructure that enables efficient metadata management and data sharing while complying with European regulations such as GDPR. The GHGA serves as the German node within the Federated European Genome-Phenome Archive (FEGA)^20^, a network designed to enable secure and jurisdictionally compliant sharing of sensitive genomic and phenotypic data across national borders. GHGA aims to establish modern research data management across academic disciplines through systematic data organization, long-term storage, backup, and accessibility.

Although metadata models for biomedical omics data exist, GHGA decided to develop its own EGA-based metadata framework to tackle the limitations mentioned previously and to ensure a scalable implementation that can be adapted to upcoming technologies, whether experimental (e.g., spatial omics, multi omics) or analytical (e.g., standardized pipelines, explorative approaches, machine learning). GHGA adopts an EGA-compatible metadata model to ensure interoperability in the FEGA context and the framework is designed to be operational within the German research and governance context. The model initially focused on cancer and rare diseases, but its design supports submissions from studies across all disease domains. This flexibility is achieved through enhancements informed by comparisons with domain-specific models (e.g. European Joint Programme on Rare Diseases (EJP-RD)^21^, International Cancer Genome Consortium-Accelerating Research in Genomic Oncology (ICGC-ARGO)^22^), disease-agnostic models (European Nucleotide Archive (ENA)^23^, database of Genotypes and Phenotypes (dbGaP)^24^, ClinVar^25^), experiment-specific metadata standards (minSCe)^8^, and in continuous collaboration with GHGA Data Hubs (e.g., a survey for the prototype model, regular meetings). This approach enables national and international alignment while retaining independence of specific methods or applications.

To assess interoperability and completeness, we conducted a crosswalk analysis comparing GHGA with four established metadata models in human genomics. The crosswalk includes the current EGA Submitter API model and the future EGA metadata model, which is currently under development. Further, we compared the ISA-tab serialized model^26^ and the metadata model developed and implemented by FAIR Genomes Netherlands^27^. The crosswalk is focused on the information content of the respective schemas instead of the entity relations or the captured vocabulary. We aim to identify interoperable fields across the frameworks that can be used to define a consensus on the information necessary to describe human omics data. This work builds on and extends MINSEQE^9^, an established minimum information standard for high-throughput sequencing experiments. The comparison with the selected models also allows us to identify possible information gaps. Ultimately, the definition of shared metadata across repositories drives interoperability in cross-consortia settings and is an important step in breaking down data silos.

### GHGA metadata model

**Fig. 1:**
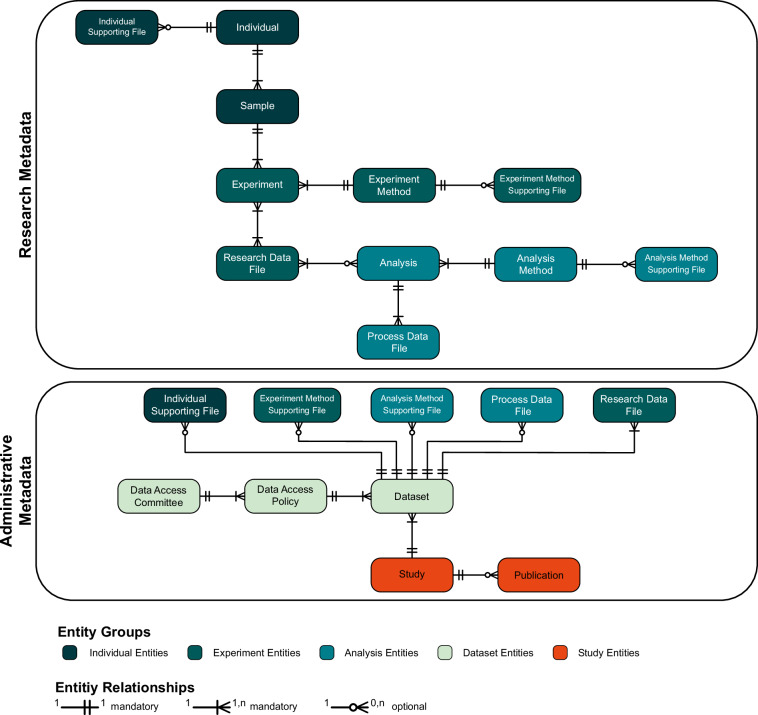
Overview of the GHGA v2 Metadata Model. The overview includes 16 schema classes, their cardinality, and number of properties. Classes are colored based on their classification in the workflow of a high-throughput sequencing experiment (Individual entities: dark green, Experiment entities: middle green, Analysis entities: blue green, Dataset entities: light green, Study entities: bright orange).

The GHGA metadata model comprises 16 entities and a total of 161 properties. Conceptually, the entities can be grouped into Research Metadata and Administrative Metadata, based on the information that is being captured within each group. As indicated in Fig. 1, five subgroups emerge within the two categories, reflecting the different parts of an omics experiment (dark green: Individual / Sample, middle green: Experiment, blue green: Analysis, light green: Dataset, bright orange: Study). Entities and properties are designed to capture non-personal metadata, which is not subject to the GDPR (Recital 26^28^). Additionally, they cover necessary information to make the process of data generation reproducible and allow data findability.

In addition to being assigned to specific classes, properties in the schema can also be categorized based on their purpose within the model. On a broad scale, we differentiate between properties that are needed for relational data model functionality and class linkage (*‘alias’*), properties to ensure FEGA compatibility (*‘ega accession’*), properties needed for ontology term validation (e.g., *‘diagnosis ids’*), indicators whether files are included in a submission (*‘included in submission’*), standardized properties to submit metadata for findable and reusable datasets, and custom properties to capture any additional, non-standardized information (*‘attributes’*).

### Research metadata

Structurally, the metadata model mirrors the design of a typical wet lab experiment, following a bottom-up approach that begins with the *Individual* — the subject of the study. Each *Individual* is connected to one or more *Sample(s)* through a one-to-many relationship, reflecting the experimental reality where multiple samples can be derived from a single individual. The *Sample* is defined as the entity that is used for conducting an omics experiment but also includes non-required details about the biospecimen it was derived from. This design allows defining biospecimen-specific details while keeping the model simple. One *Sample* is subject to one or more *Experiments*, where each *Experiment* links to exactly one *Experiment Method*. The *Experiment Method*, in turn, can link to one or more *Experiment(s)*, indicating multiple experiments can follow the same experimental protocol.

Each *Experiment* is connected to one or more *Research Data Files* capturing the raw files, such as FASTQ files or BAM files containing unmapped reads. The linkage from *Research Data File* to *Experiment* follows a many-to-many relationship, allowing to also represent multiplexed files, an approach commonly used in single cell sequencing experiments^29^.

Further processing of the *Research Data File* necessitates linking the latter to the *Analysis* class, however, submitting processed data is optional, as indicated by the zero-to-many cardinality. Whilst each *Analysis* follows exactly one *Analysis Method*, it produces one or more *Process Data File(s)*. These contain processed data, such as variant calls (VCF files), alignment maps (BAM or CRAM), or any other file format originating from downstream bioinformatic data processing. Contrary to the many-to-many relationships between *Research Data File* and *Experiment*, *Process Data File* and *Analysis* are linked via a one-to-many relationship, indicating that one *Analysis* produces one or more *Process Data Files*, while each *Process Data File* originates from only one *Analysis*.

To capture additional, non-standardized information, submitters are encouraged to include supporting files, which are supported for the *Individual* (*Individual Supporting File*), the *Experiment Method* (*Experiment Supporting File*) and the *Analysis Method* (*Analysis Method Supporting File*).

### Administrative metadata

All five file types (*Research Data File*, *Processed Data File*, *Individual Supporting File*, *Experiment Method Supporting File*, *Analysis Method Supporting File*) must be linked to a *Dataset*. Submitters have the flexibility to define *Dataset(s)* according to their needs, for example, by distinguishing between raw and processed data, different experimental methods (such as various sequencing techniques), or participant groupings (e.g., case vs. control, diseased vs. healthy). Each *Dataset* is governed under one *Data Access Policy*, however one *Data Access Policy* can refer to multiple *Dataset(s)*. Similarly, each *Data Access Policy* is governed by one *Data Access Committee*, which can manage multiple policies. Further, each *Dataset* is linked to one *Study* and one *Study* must contain one or more *Dataset*. If available, submitters can indicate any related publications using the *Publication* class.

### Property status

As with entities, properties in the GHGA schema are categorized as required, recommended, or optional. This classification is based on their importance for database functionality, data discoverability, and reusability. It was defined in alignment with the EGA metadata model and considering user feedback.

Across all 16 classes, a total of 88 properties is required. These mainly cover class aliases, which are necessary to link the data, as well as titles, names, and descriptions, which are presented on the data portal. Further, properties that ensure data reusability, such as the individual’s sex and age, information about the sequencing library and instrument, and the bioinformatics pipeline used to process data, ensure that data is reusable for other purposes. Administrative metadata, such as Data Use Ontology (DUO)^30^ terms and Data Access Committee emails, are necessary for data access management.

Recommended properties carry additional information that increases the findability and reusability of a dataset. Accordingly, the majority of the 36 recommended properties can be found in the *Sample* and *Experiment Method* classes, where they capture details about the biospecimen that was used to generate the *Sample*, or further information about the sequencing library kit and the flow cell that was used for sequencing. The *Individual* and *Analysis Method* classes also carry recommended properties, such as the phenotypic features and diagnoses associated with an *Individual*, or details about the workflow and reference annotation that were used to process data.

Finally, the metadata schema contains 37 optional properties. These cover geographical and ancestry information about the data subject, external references for biosamples, or GHGA-specific fields, such as EGA-accessions.

Standardization is achieved by controlling properties by either utilizing existing and widely used ontologies, or defining custom-controlled vocabularies. An overview of all defined vocabularies can be found in the schema (https://github.com/ghga-de/ghga-metadata-schema/blob/2.2.0/src/schema/submission.yaml#L1410) or the GHGA data dictionary (https://docs.ghga.de/metadata/data_dictionary/00_overview/). A total of 66 properties are controlled, where 44 only accept values from defined vocabularies. These word lists are either aliases to ensure proper linkage, or were generated following EGA vocabularies, Laboratory Information Management System (LIMS) readouts from GHGA data hubs, and other domain experts. Further, 14 properties are controlled using ontologies, namely the Human Phenotype Ontology (HPO)^31^ for phenotypic features, ICD-10 for diagnoses (https://icd.who.int/browse10/2019/en), the National Cancer Institute Thesaurus (NCIt)^32^ for the geographic region, Human Ancestry Ontology (HANCESTRO)^33^ for the ancestry, BRENDA tissue ontology (BTO)^34^ for the biospecimen tissue, and DUO to code data access permissions and modifiers. The remaining eight properties are controlled by the definition of the slot type as a Boolean or an integer.

### Architectural and legal framework

#### Architectural framework and submission tools

The GHGA metadata model is implemented in the Linked Data Modelling Language (LinkML)^35^. The model is rooted in the *Submission* class. For every class an identified slot (primary key) named *‘alias’* is defined which can then be used to create references across entities. Users are enabled to extend the given model with additional metadata items through a generic slot definition that consists of key-value pairs (*‘attribute’*). Repetitive slot definitions across the model are reduced by utilizing Mixins.

GHGA offers data submitters an Excel Workbook, auto-generated from the schema and serving as a user-facing submission template, providing a human-readable format that is better suited for users without a programming background. The direct submission of metadata using a JSON is also possible. Prior to metadata submission to GHGA’s downstream backend tools, the metadata is processed with command-line tools developed in-house, ghga-transpiler (https://github.com/ghga-de/ghga-transpiler) and ghga-validator (https://github.com/ghga-de/ghga-validator).

**Fig. 2:**
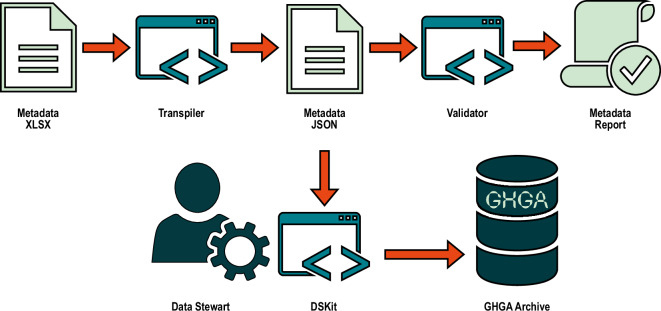
Flow of metadata validation in the GHGA Architecture. Incoming metadata excel sheets are transpiled into JSON-format using the GHGA Transpiler. The GHGA Validator validates the JSON against the GHGA metadata model and generates a report in JSON format. A data steward forwards the validated metadata JSON to the GHGA Archive using the Data Steward Kit (DSKit).

As shown in Fig. 2, the transpiler tool converts the submission Excel workbook into a JSON file, a machine-readable data format. It is agnostic to the workbook structure but relies on a configuration file to import the workbook settings. The transpilation routine includes auto-processing of data values into model conforming data types and formats using built-in functions specific to GHGA metadata models. After transpilation, the validator tool checks whether the metadata is valid based on the GHGA metadata model. This step includes structural validation, checks for completeness, and verification of the uniqueness of aliases. The validator accepts the metadata as JSON and the metadata model as YAML and generates a report in JSON format, which is user-friendly and easy to interpret.

#### Privacy considerations

A key challenge in developing an effective metadata model is recognizing that metadata can qualify as personal data under Art. 4 GDPR. Personal data relates to identifiable, living individuals, even if identification is only possible through combining data with other sources. This presents a dilemma: metadata must be detailed enough to support FAIR principles and aid research, yet it must not reveal the identities of Data Subjects, which could breach Art. 5 GDPR.

In GHGA’s case, metadata like sex, age, ancestry, and diagnosis helps researchers assess data relevance before applying for access. However, rare combinations of such attributes could make individuals identifiable. For instance, a 24-year-old male from a minority group with breast cancer is highly identifiable due to the rarity of that profile.

A report by Weichert and Schuler from Netzwerk Datenschutzexpertise showed how combining variables such as sex, postcode, and diagnosis can lead to patient identification, even in anonymised hospital data^36^. This underlines the need to treat indirectly identifying metadata as personal data if linkage is reasonably likely – a standard clarified in Recital 26 GDPR, which considers cost, time, and technology involved in re-identification.

In developing the GHGA Data Portal, ensuring metadata was non-personal was critical, especially given the inclusion of health-related data, which falls under special category data (Art. 9 GDPR). GHGA conducted a Privacy Impact Assessment to evaluate identifiability risks. It revealed that demographic variables in the GHGA model overlapped with those in the hospital data identified by Weichert and Schuler as leading to potential re-identification. However, a number of key changes have been made:

1. Low-level geographic information remains private
  The inclusion of a postcode or other forms of low-level geographic information has the effect of reducing the size of the possible population within which the Data Subject exists, as well as narrowing down the geographic area in which a Data Intruder would have to search for further information. Germany has a population of approximately 84 million people, whereas a postcode area will usually have a population of a few thousand people. Assuming that incidence rates are consistent across postcode areas, the number of cases in any postcode area is likely to be low, particularly for less common phenotypes. Data deposited at GHGA will usually have been generated in Germany, but no further geographic information is presented in the public metadata.
2. Banded age
  Rather than presenting the Data Subjects’ exact ages or dates of birth, the GHGA Metadata Model uses banded ages. For example, a Data Subject’s age recorded in categories of ten years: age 20–30, rather than 24 years. A similar approach was taken in the hospital data analysed by Weichert and Schuler^36^. However, in the analysed hospital data, age was banded to five-year increments, which Weichert and Schuler considered to be insufficient.
3. Amalgamated diagnoses
  The GHGA Data Portal does not utilise the full ICD-10 code, and instead amalgamates different phenotypes together that share the same top-level ICD-10 code (e.g., U31.0, U31.1, U31.2 become U31). By only utilising the letter and two numbers, the ability to isolate rarer variations of a phenotype, a likely vector of an attack, is reduced, and so the level of protection for any individual Data Subject is increased. This approach was not used in the hospital data analysed by Weichert and Schuler.

As a result of these protections, despite the similarity of certain fields with the hospital data analysed by Weichert and Schuler, the metadata contained with the GHGA Data Portal can be considered to be non-personal within the meaning of Recital 26 GDPR. To support this classification, GHGA produced a Privacy Impact Assessment to examine whether the metadata captured in its model could reasonably be regarded as non-personal data, taking into account its expected contents and the additional data protection measures in place. As the model itself does not contain metadata, it is not possible to provide formal privacy guarantees, such as assurances regarding compliance with k-anonymity, since such tests require the presence of actual metadata. Instead, the GHGA metadata model has been deliberately designed to reduce the likelihood of such violations, which, combined with GHGA’s broader data protection measures, led to the conclusion that the publicly displayed metadata can be reasonably considered non-personal. The metadata remains at the individual-level, to better enable discovery, but the model has sufficient reductions in precision that the effort required to identify a Data Subject would be unreasonable. In this way, it strikes a balance between findability and data protection.

## Results

The full GHGA metadata model^37^ was used as the basis to perform the semantic crosswalk analysis to selected biomedical models. For each of the other models, the latest schema versions were retrieved from their respective GitHub repository or the EGA Submission API. Properties within each model were included or excluded based on the mapping rules clarified in the methodology. During the mapping analysis, instances were identified where a single property corresponded to multiple fields in the target model. Plots will depict only one connection to enhance visual representation.

### Forward mapping between GHGA and related models

The crosswalk analysis between the GHGA metadata model and four external frameworks revealed generally consistent property coverage. After applying mapping rules, 96 GHGA properties were analyzed. FAIR Genomes achieved the most matches (50), followed by EGA (42), ISA-tab (36), and the EGA Submission API (36). Most correspondences were exact matches, while EGA-based models showed more lexical alignments. Statistical analyses found no significant association between model type or GHGA class and mappability (p > 0.3). Coverage varied across GHGA classes, with *Study* and *Analysis* showing the highest alignment (up to 88%), while *Experiment Method* and *Research Data File* showed lower rates. All GHGA classes had at least one equivalent property in external models, confirming the model’s interoperability. Additional statistical data and full mappings are provided in the Supplement.

### Identification of consensus omics metadata properties

**Fig. 3:**
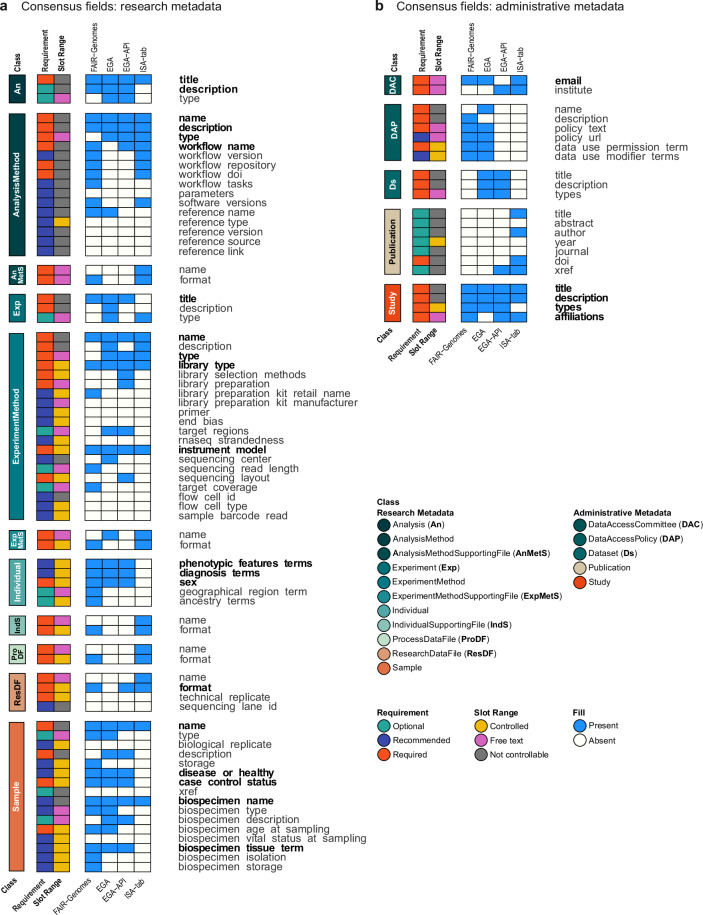
Property presence / absence matrix based on the GHGA Metadata model. Summary of the forward mapping between GHGA and FAIR Genomes, EGA, EGA Submission API and ISA-tab divided into research metadata (**a**) and administrative metadata (**b**). Each row represents one property in the GHGA model. Columns indicate the property annotations class, requirement (required: orange, recommended: purple, optional: turquoise) and range (controlled: yellow, free text: pink, not controllable: grey). The presence of equivalent properties in the compared model is indicated with blue tiles, while white tiles show that a model did not have a matching property. GHGA model properties that were mappable to minimum three models are highlighted in bold text.

Based on the mapping analyses performed between the GHGA model and the EGA Submission API model (Fig. S1a), FAIR Genomes metadata model (Fig. S1c), the EGA model (Fig. S2a) and the ISA-tab model (Fig. S2c), we identified overlapping properties (Fig. 3a,b). A presence-absence matrix was derived from the mappings to indicate recorded connections for each property in the GHGA metadata model. A property was declared as “common” if it was present in at least three out of the four models that were compared to the GHGA model.

Out of 96 total properties included in the mapping, 25 were identified as common fields of information, spread across nine classes in the GHGA model. The most overlapping fields were identified for the *Sample* (five properties), followed by *Study, Experiment Method*, *Analysis* (four each), *Individual* (three), *Analysis* (two), and lastly *Data Access Committee* and *Experiment* with one common property each. The three supplementary file types, *Data Access Policy*, *Dataset* and *Publication* had no common fields.

While 10 properties were present in all five metadata frameworks, 15 were present in GHGA and three other models. ISA-tab had the most missing fields (nine), followed by EGA with three missing properties and FAIR Genomes with two, whereas only one field was absent in the EGA Submission API model.

The identified common properties can be classified based on the possibility to control data entries using defined vocabularies, ontologies, or regular expressions, which is possible for 15 fields. These fields capture standardizable information about a genomics experiment, namely methodological classifications (*Analysis Method ‘type’*, *Experiment Method ‘type’*, *Study ‘types’*), procedural details (*Experiment Method ‘library type’*, *Experiment Method ‘instrument model’*) and file format information (*Research Data File ‘format’*). Further controllable fields capture information to describe samples (*Sample ‘disease or healthy’*, *‘case control status’*, *‘biospecimen tissue term’*) or sample donor characteristics (*Individual ‘phenotypic features terms’*, *‘diagnosis terms’*, *‘sex’*). Additionally, information about study affiliations (*Study ‘affiliations’*) and contact details (*DAC ‘email’*) are among the consensus properties.

The remaining 10 consensus fields capture information to uniquely identify entities within one study (*‘name’* or *‘title’*) or to describe them for data reusers (*‘description’*). Due to their heterogeneous nature, they cannot be controlled using vocabularies, ontologies or pattern identification mechanisms. Properties serving identification and description purposes are present for the *Analysis* (*‘title’*, *‘description’*), *Analysis Method* (*‘name’*, *‘description’*, *‘workflow name’*), *Experiment* (*‘title’*), *Experiment Method* (*‘name’*), *Sample* (*‘name’*, *‘biospecimen name’*) and the *Study* (*‘title’*, *‘description’*).

All identified common properties, except for *Analysis ‘description’*, are either required or recommended in the GHGA metadata schema. A majority of 19 consensus fields are required. Among these are fields to identify and describe entities, methodological type and procedural specifications, as well as contact and affiliation details. Further, *Individual ‘sex’* and *Sample ‘case control status’* are required information. The recommended properties are amassed in the *Sample* and *Individual* entities and capture information about a person’s phenotypic features or diagnoses, and a sample’s disease or healthy status, as well as a biospecimen identifier and the tissue.

### Backward mapping from related models to GHGA

Before the crosswalk analysis, properties were filtered according to mapping rules, leaving between 30% and 45% of properties per model for comparison. EGA and its Submission API showed the strongest overlap with GHGA (48 and 35 matched properties, respectively), while FAIR Genomes (33) and ISA-tab (25) displayed lower alignment. Most connections were exact matches, with only a few narrow or unmatched properties observed. EGA Submission API achieved the fewest unmatched entries (two) and the highest mean coverage with GHGA (97%), followed by EGA (79%), ISA-tab (71%), and FAIR Genomes (40%). Statistical testing confirmed a significant relationship between model and property mappability (p < 0.001). GHGA classes such as *Analysis Method*, *Experiment Method*, *Sample*, and *Study* were represented in all models, while auxiliary files had no equivalents. Median mappability of common classes was highest for EGA-API and ISA-tab, underscoring their closer conceptual alignment with GHGA.

### Knowledge gap identification

**Fig. 4:**
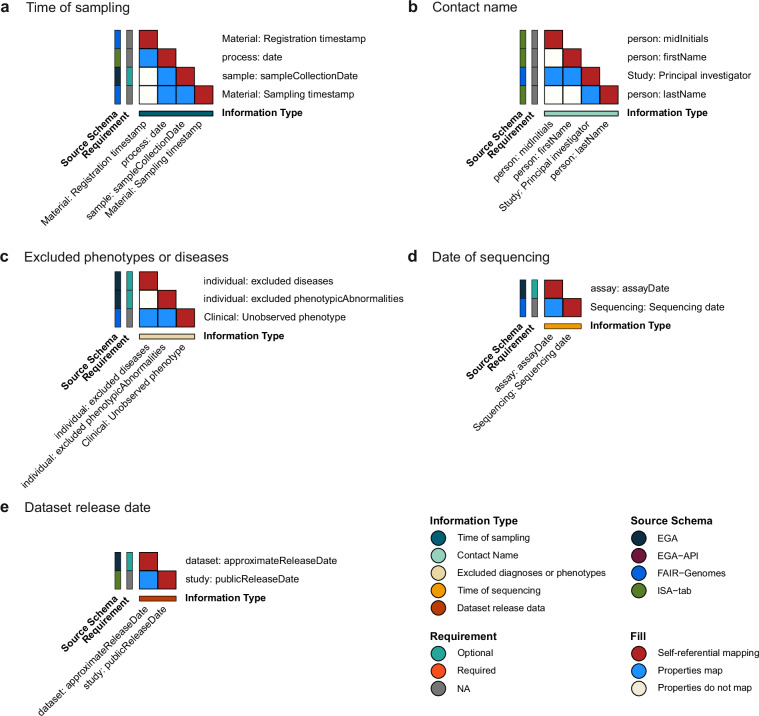
Overview of information types not present in the GHGA model. Mapping matrices are grouped based on the information type captured by the properties. Properties are annotated with their respective source schema (EGA: dark blue, EGA Submission API: dark red, FAIR Genomes: royal blue, ISA-tab: green) and requirement (required: orange, optional: turquoise, not applicable: grey). Matching of properties is indicated with blue tiles. White tiles depict that properties do not map. Red tiles show self-referential mapping.

Out of the 232 properties analyzed across the four related metadata models, 91 properties could not be mapped to the GHGA model. To identify potential knowledge gaps, we examined these unmapped properties by cross-mapping them and organizing them into information complexes based on their content (Fig. 4). This analysis revealed that no single information complex was present and required across all four related models while simultaneously being absent in the GHGA model. Additionally, none of the properties in the absent information groups were designated as required in their respective schemas.

However, we identified one information complex that was mappable across three of the four models. These are information about the time of sampling, which is present in EGA (*Sample ‘collection date’*), FAIR Genomes (*Material ‘Sampling timestamp’* and *‘Registration timestamp’*) and ISA-tab (*Process ‘date’*) (Fig. 4a). While FAIR Genomes and ISA-tab have no information on the requirement status of their properties, this type of information is optional in the EGA schema. FAIR Genomes’ *Material ‘Registration timestamp’* only maps to ISA-tab *process ‘date’* but not to the other two properties in this group.

The other four information complexes stretch across two of the four models. Figure 4b depicts properties defining information about a contact name. These properties are present in the ISA-tab model (*Person ‘midinitials’*, *‘firstName*, *‘lastName’*) and FAIR Genomes (*Study ‘Principal Investigator’*). The latter property maps to all three ISA-tab properties, whereas there is no recorded mapping among the ISA-tab properties.

Further, the GHGA model does not capture information about excluded diseases or phenotypes, as well as the date of sequencing. This information is collected both in the EGA (*Individual ‘excluded diseases’*, *‘excluded phenotypicAbnormalities’*; *Assay ‘assayDate’*, respectively) and FAIR Genomes (*Clinical ‘unobserved phenotype’*; *Sequencing ‘sequencing date’*, respectively) models (Fig. 4c, d). In both cases, this information is optional in the EGA schema. Lastly, EGA and ISA-tab both capture information about the dataset release date (EGA: *Dataset ‘approximate release date’*, ISA-tab: *Study ‘public release date’*). Similar to the previous fields, submitting this property is optional (Fig. 4e).

## Discussion

The GHGA metadata model provides a flexible and extensible foundation for describing biomedical and genomics data across diverse research contexts. Its adaptable design extends existing models, such as the EGA model, while being developed in close collaboration with GHGA stakeholders and aligned with international standards. GHGA ensures interoperability with major international genomics initiatives by adopting single properties from ICGC-ARGO and dbGaP and by integrating controlled vocabularies from ontologies such as ICD-10, HPO, and NCIt. The model incorporates domain conventions from initiatives including EJP-RD, distinguishing phenotype from diagnosis, and integrates selected properties from ENA, ICGC ARGO, GDC, and dbGaP to support semantic interoperability. GA4GH standards such as DUO codes and crypt4GH are embedded within the framework, while Phenopackets can be submitted as supplementary files to enhance phenotype representation.

The crosswalk analysis between GHGA as source and EGA Submission API, EGA, FAIR Genomes, and ISA-tab as target models allowed us to define a consensus to collect information about experiments in the field of biomedical omics. Forward mapping (from GHGA to the related models) and backward mapping (from the related models to GHGA) proved effective for identifying shared information, uncovering potential knowledge gaps, and highlighting structural differences across the selected models.

The high number of exact lexical matches in both forward and backward mapping directions highlights the overall similarity between the five compared models and alignment to domain best practices. Only GHGA *Sample ‘biospecimen name’* and *Experiment Method ‘target regions’* were repeatedly mappable based on background knowledge, which was the case in the crosswalks to the EGA Submission API and EGA models.

### Forward mapping reveals cross-model alignments and similarities

As expected, mapping the GHGA model to four related metadata models revealed no significant differences in overall mappability, confirming its broad interoperability. While GHGA was initially aligned with the EGA Submission API, it has since expanded to support complex biomedical omics use cases. The comparable mappability rates among models reflect this balanced design, enabling integration across diverse standards.

Lower alignment observed for the EGA-API can be attributed to its smaller and more technically focused scope, whereas the more general ISA-tab model demonstrated broader compatibility due to its flexible, less domain-specific classes. EGA and FAIR Genomes showed similarly high match rates but differed in emphasis: EGA overlapped with GHGA in technical domains such as analysis and experiment metadata, while FAIR Genomes aligned more closely in administrative domains like data access and study information.

Several GHGA properties, particularly those within the *Experiment Method* and other highly specialized classes, did not align with external models. These fields capture detailed experimental metadata such as sequencing platform or library kit that, while optional, can help trace potential batch effects and ensure more reproducible analyses.

### Defining a consensus for biomedical omics metadata models

All five models cover basic information about the overarching study, sample characteristics, such as sex and phenotype, the process of sequencing, such as library and sequencer, downstream analysis workflows, and contact details. Further, all models in the crosswalk store information about the file type or format that was produced during the experiment. Among these, several fields are controllable using either defined vocabularies or ontology validation, allowing for standardization across metadata models. The identified consensus aligns with other widely used data portals that are not included in the analysis, such as GEO^38,39^ or ArrayExpress^40^. However, the information content in both differs and is not fully standardized, requiring metadata curation efforts prior to data re-use^38,39^.

### Consensus blind spots

The information captured in the consensus can be utilized for basic re-use cases and machine-learning approaches. However, it does not collect enough metadata for in-depth analyses integrating multiple datasets. Next to detailed clinical information, the consensus lacks standardized information about library preparation protocols, preprocessing pipelines and file formats of processed data files. While protocols in clinical routine scenarios might not differ from manufacturer recommendations, academic experiments often divert from standardized protocols, testing new techniques or combinations of single library preparation workflow elements. For reasons of reusability and interpretability, it is necessary to record this information. Although the selected frameworks focus on bulk approaches, the GHGA model is also capable of capturing metadata of transcriptomics experiments on the single cell level. Since this approach massively increases the granularity of observable differences within a dataset, more details about the experiment have to be collected in order to correctly identify sources of confounders, for example the storage temperature^41,42^. This detailed information cannot be found in the consensus.

### Applying the consensus to MINSEQE and beyond

Although additional information beyond the basic necessary fields is not present in the consensus, the comparison of five models serving different use cases solidifies the importance of the identified common fields and offers a resource to define metadata models in alignment with established standards. Additionally, the consensus fully aligns with MINSEQE^9^, the Minimum Information about a high-throughput Sequencing Experiment, which mandates descriptions of five elements of an omics experiment. Sample details, sequence read data, information about the study or experiment, and protocols are directly covered in the consensus fields, while processed or summary data are not mentioned in the common fields. However, all five compared models offer linking properties to capture both sequencing and processed or summary files. File identifiers are not explicitly part of the standardized information captured in the semantic consensus fields.

Beyond MINSEQE the consensus eases alignment to other health-related domains, such as biobanking standards or clinical trial registries. On the national level, the study-level consensus fields are mappable to the mandatory items of the NFDI4Health metadata schema core module^43^. Internationally, the consensus properties allow a high-level mapping to MIABIS (descriptions of samples, datasets, research resources, and collection)^44^. These alignments enable cross-domain linking in the context of studies and increase the FAIRness of the deposited data.

### Backward mapping reveals significant model differences

The backwards mapping of the four related models to the GHGA model revealed a significant difference with regards to general mappability. The EGA Submission API and ISA-tab models had the highest mappability rates. While none of the missing fields are universally required, several, such as sampling or sequencing date, could be prioritized in future iterations, following a privacy assessment of the combinations of dates and age ranges.

Non-mappable properties in the ISA-tab model can be separated into two distinct information groups: dates, such as *Investigation ‘submissionDate’*, and personal information, such as *person ‘lastName’* and excluded phenotypes or diseases. Further, the *Material* class (*‘name’* and *‘type’*) and *Publication ‘status’* were not mappable to the GHGA model. The GHGA metadata model operates under the assumption of anonymity, excluding any potentially identifiable personal information, such as individual names or personal addresses. Although it does not carry personal information, excluded phenotypes and excluded diseases can serve as quasi-identifiers in combination with other metadata attributes and are therefore excluded from the model. ISA-tab defines the *Material* class to describe any consumables during the experimental process, and *Sample* as specification of the *Material* class (https://isa-specs.readthedocs.io/en/latest/isamodel.html). The ISA-tab *Material* class can therefore be linked to the GHGA *Sample*, but as both models carry a dedicated *Sample* class, linking only between these specialized classes is semantically more appropriate. Since ISA-tab’s use case is to offer a serialized model that can be adapted to many different use cases by customizing *Comment*, *Factor*, and different attribute and value classes, the content of the GHGA metadata model can also be encoded there.

The Kruskal-Wallis test revealed a significant difference of the mappability rates of common classes among the four models. This difference was most pronounced in the mapping of FAIR Genomes to GHGA, whose median mappability rate was 0.46. This finding is in line with FAIR Genomes’ use-case. While all the other analysed models are designed to capture study-centric information about an omics experiment freely designable by the researcher, FAIR Genomes collects data about genomics approaches in a precision medicine setting, using a human-centric relationship modelling. As such, this model includes detailed information about consent forms and dates, as well as further clinical information about the individual under study. The information content of the FAIR Genomes model is comparable to Genomdatenverordnung (GenDV, https://www.gesetze-im-internet.de/englisch_gendv/englisch_gendv.html), specifying type and scope of data collected, stored, and processed under the Modellprojekt Genomsequenzierung^45^. The GHGA model is not designed to capture consent form information, especially not on the individual level, but important information from the consent form for one study might be included in the data access policy text. Still, details about both the consent form signed by the individual and clinical or demographic data can be submitted to GHGA using the *Individual Supplementary File*. Operating under the assumption of anonymity under the GDPR made it necessary to select the most common confounders of data analyses in the field of biomedical research (disease, sex, age, phenotype, country of residence, ancestry) on a broader level (banded age, low-level geographic information, aggregated diagnosis), leaving out other information of interest, such as comorbidities, year of birth, age at disease onset, medication, or information that would most likely be limited due to data minimisation according to Art. 5c GDPR^28^, such as gender identity data. In the same context, model design choices were affected by similar privacy considerations. Patient-centric models require stronger privacy safeguards when metadata are publicly displayed or searchable, as they link more data to individuals and increase re-identification risk across studies. They also demand networked data architectures rather than isolated submissions. While such designs exist (for example, the 4D nucleome project^46^), GHGA adopted a study-centric approach aligned with FEGA and common omics repositories like EGA and GEO to ensure feasibility and familiarity for submitters.

### GHGA covers all mandatory metadata fields

None of the properties that were absent in GHGA were present in all four related models. We identified information groups common across two to three compared models, highlighting room for improvement in future GHGA model releases. Nevertheless, three out of five information types can currently be submitted to the GHGA infrastructure using custom attributes for *Sample* (Time of sampling), *Experiment Method* (Date of sequencing), and *Study* (Dataset release date). Contact names and excluded diseases or phenotypes on the level of individual patients were deliberately left out of the GHGA metadata model to comply with the assumption of anonymous data collection. The *Individual Supplementary File* allows to append details about individuals to the dataset without this information being shown in the GHGA data portal.

Of the four models, only EGA API and EGA differentiate between required and optional properties. While the GHGA metadata schema covers all required slots in the EGA Submission API model, *Individual ‘organismDescriptor’*, as well as *Sample ‘organismDescriptor’* and *‘sampleGroupBoolean’* of the EGA mandatory fields are not mappable to GHGA. In GHGA, both EGA *‘organismDescriptor’* instances default to ‘NCBITaxon:9606’ and ‘homo sapiens’^47^ because GHGA only collects data from human individuals. In the EGA model, *‘sampleGroupBoolean’* is used to indicate whether a sample object corresponds to an individual sample or a sample group. The GHGA model does not differentiate sample grouping on this level but it is possible to infer the distinction programmatically by counting unique occurrences of *‘individual alias’*, used to link samples to individuals, in the *Sample* class. In conclusion, all required fields can be filled using the GHGA model, although directly corresponding fields are not always present in the model.

### Conclusion and future perspectives

As discussed in the previous section the GHGA metadata model is suitable for many use cases with regards to common sequencing approaches (WGS, WES, bulk and single-cell RNAseq) in clinical settings or biomedical studies. It is mappable to relevant European initiatives (EGA, FAIR Genomes) and models that are implemented on a global scale (ISA-tab). The identification of a consensus across all five models in the crosswalk emphasizes the importance of metadata interoperability and the alignment to accepted standards, such as MINSEQE for high-throughput or minSCe^8^ for single-cell sequencing experiments. While a comparison of accepted controlled vocabularies or ontologies was not part of the analysis presented here, standardization across models also needs to take vocabularies into account to be fully interoperable^48^. As in all databases that operate on ontology implementation, an important aspect that needs a solution is the versioning of ontologies and the subsequent translation to metadata model values that have already been stored in the database^49,50^.

Comparing our model with other models in the field allowed us to identify possible gaps in the information collected by the metadata model. While none of the identified additional fields are present in all of the compared frameworks, underlining the overall completeness of the GHGA metadata model, further adjustments could include a sampling date in the *Sample* class and the sequencing date in the *Experiment Method*.

The GHGA metadata model in its current version was developed to capture research and administrative metadata mostly from bulk DNA or RNA sequencing experiments. As the neighboring fields are steadily evolving, it will become necessary to develop the model further into the direction of other state-of-the-art techniques, such as proteomics^51^, ATAC (Assay for Transposase-Accessible Chromatin) or spatial omics^52^. All of these methods come with unique data and metadata requirements; for example the connection of sequencing data and imaging data in spatial omics, or a combination of multiple omics layers. The increasing complexity of experimental and analytical approaches, in combination with recent technical developments in the field of federated data processing and data visiting, such as trusted research environments or swarm learning^53^, necessitate a progression to increasingly flexible metadata models that can handle even more diverse use cases. The GHGA metadata model offers a sound basis, both on a technical and legal level, for further advancements and illustrates how FAIR sharing of omics data and GDPR-compliance can go hand in hand.

Furthermore, the current metadata model has a great potential to fully harness the emerging technologies in healthcare. Future metadata frameworks must evolve towards greater flexibility, adaptability, and interoperability, incorporating AI-driven schema evolution, supporting decentralized architectures, and enabling seamless integration of multimodal datasets. Addressing these challenges is imperative to ensuring that metadata remains a robust foundation for next-generation healthcare innovations.

## Methods

Calculations and visualizations were performed using R^54^ and the R packages *dplyr*^55^, *tidyr*^56^*, ggplot2*^57^, *circlize*^58^, and *pheatmap*^59^.

### Semantic mapping

Schema representations from EGA (https://github.com/EbiEga/ega-metadata-schema/tree/b82f410b184084a326d7eaffd1feb729f46676a1/schemas), the EGA Submission API v3.0 (https://submission.ega-archive.org/api/spec/#/), the FAIR Genomes (https://github.com/fairgenomes/fairgenomes-semantic-model/blob/e17e51e4b79f8a1498cefaf305c8cb8cf8f6340e/fair-genomes.yml), ISA-tab (https://github.com/ISA-tools/isa-api/tree/95a788c50a0b908118c2d16ad342478d3f5ef064/isatools/model), and GHGA (https://github.com/ghga-de/ghga-metadata-schema/blob/2.2.0/src/schema/submission.yaml) were converted into a long tabular format. Mappings between source and target properties were recorded in accordance with the SSSOM standard^60^. Connections were labelled using the Semantic Mapping Vocabulary (semapv) introduced by SSSOM^60^ and Simple Knowledge Organisation System (SKOS) vocabulary (https://www.w3.org/TR/skos-reference/). Mapping predicates were defined as one of *exactMatch* or *closeMatch*, indicating that object and subject can be used interchangeably across a wide range of (*exactMatch*) or some (*closeMatch*) information retrieval applications, *broadMatch* or *narrowMatch*, in which the object is either a narrower or a broader concept than the subject. Mapping justifications were defined using *lexicalMatch* and *Background Knowledge Based Mapping* (*BKBM*). Properties with no corresponding match were recorded as *noMatch*.

Properties in the models were mapped based on their description, ensuring that they capture the same information, even though property naming conventions might differ. In cases where descriptions or property names were unclear, we compared controlled vocabularies, if they were available. Properties were always mapped to a property in the target model; mapping properties to classes only (e.g., EGA *DAC ‘objectTitle’* to GHGA *Data Access Committee*) was not possible. All mappings are human-curated.

Several properties were excluded from the mappings. These are non-defined properties, such as GHGA *‘attributes’*, ISA *‘comments’* or EGA API *‘extra attributes’*, linking properties, such as *‘aliases’* or entity IDs, and framework-specific properties that are needed for schema functionality, such as GHGA *‘ega accessions’* and ontology term IDs, or EGA API provisional IDs. By excluding these properties, we ensured that the mapping focuses on the fully standardized model parts. This allowed the comparison of the information content irrespective of the model structure or entity relationships.

### Information gap identification

To identify gaps in the GHGA model, properties that were present in the other four models (related models) but absent in the GHGA model were collected and mapped amongst the four compared models. The cross-model mapping only included those properties that were not present in the GHGA model. Mappable properties were grouped based on the captured information to investigate knowledge gaps within the GHGA model.

### Coverage calculations and statistics

Mean coverage percentages were calculated for each class (*P*_*C*_) and model (*P*_*i*_) in both mapping directions (forward and backward) based on the mappability rate per class. The value is determined by dividing the number of mappable properties per source model class to a target model (*m*_*C,i*_) by the total number of properties in that class (*t*_*C,i*_), excluding those omitted from the analysis based on the applied mapping rules (*e*_*C,i*_) (see Eq. 1).

1 $${P}_{C,i}=\frac{m(C,i)}{t(C)\,-\,e(C)}$$

Where

- *P*: mapping percentage
- *C*: a source model class
- *m*_*C,i*_: number of mapped properties for class *C* in model *i*
- *t*_*C*_: total number of properties for class *C*
- *e*_*C*_: number of excluded properties for class *C*
  For forward mapping from GHGA to the related models, the mean coverage for each GHGA class (*P*_*C*_) was calculated by adding the mapping percentages for one class to each of the four target models (*P*_*C,i*_) and dividing the sum per class by the number of models (Eq. 2).
  2 $${\underline{P}}_{C}=\frac{1}{4}{\sum }_{i=1}^{4}{P}_{C,i}$$
  Where
- *P*: mapping percentage
- *C*: a source model class
- $i\in \{\mathrm{1,2,3,4}\}$: index of a target model
  For backward mapping, the mean coverage per model (*P*_*i*_) was calculated by summing the mapping percentage of each class within the model (*P*_*C,i*_) and dividing the sum by the number of classes in the model (Eq. 3). The calculation omitted classes that were excluded from the mapping based on the defined mapping rules.
  3 $${\underline{P}}_{i}=\frac{1}{|{n}_{i}|}{\sum }_{c\in {C}_{i}}{P}_{C,i}$$
  Where
- *P*: mapping percentage
- *C*: the set of included classes
- $i\in \{\mathrm{1,2,3,4}\}$: index of a model
- ${n}_{i}$ = ∣_*C,i*_∣: Number of included classes in model *i*

To compare mappability rates in the backwards mapping, standard classes were identified and classes in the model assigned to them. The standard classes were *Study*, *Sample source*, *Experiment* and *Analysis*. Classes included in the backwards mappability comparison were EGA API’s *StudyRequest* (*Study*), *SampleRequest* (*Sample source*), *ExperimentRequest* (*Experiment*), and *AnalysisRequest* (*Analysis*), EGA’s *Study* (*Study*), *Sample*, *Individual* (both *Sample source*), *Experiment*, *Assay* (both *Experiment*), *Protocol* and *Analysis* (both *Analysis*), FAIR Genomes’ *Study* (*Study*), *Personal*, *Clinical*, *Material* (all *Sample source*), *Sample preparation*, *Sequencing* (both *Experiment*) and *Analysis* (*Analysis*), and ISA-tab’s *Study* (*Study*), *Source*, *Sample* (both *Sample source*), *Assay* (*Experiment*), *Process* and *Protocol* (both *Analysis*).

Statistical tests were employed to test mapping differences for both mapping directions. A Pearson’s Chi-squared test was used to test whether mappability rates are independent of the compared model. Differences between class mappability rates were tested using a subsequent Kruskal-Wallis test. In the backwards mapping, the Kruskal-Wallis test was only applied to the previously defined standard classes.

## Supplementary information

Supplement: Detailed crosswalk analyses (forward and backward mapping)

## Data Availability

The complete crosswalk data is available on Zenodo^61^.

## Code availability

The code to generate figures and perform statistical evaluation is available in the GHGA Metadata Crosswalk Analysis repository (https://gitlab.dzne.de/ag-ulas/ghga-metadata-model-crosswalk-analysis).
