# Supplementary material for: Semantic alignment of the German Human Genome-Phenome Archive metadata model in Europe’s genomics field
Source: Sci Data. 2026 Feb 11;13:242. doi: 10.1038/s41597-026-06575-y (PMC12905157; doi:10.1038/s41597-026-06575-y)
Supplement: Supplementary file 1 — Supplement: Detailed crosswalk analyses (forward and backward mapping) [file 41597_2026_6575_MOESM1_ESM.pdf]

Table of contents

Forward mapping 2
EGA API 2
FAIR Genomes 3
EGA 5
Backward mapping 8
EGA API 9
FAIR Genomes 9
EGA 12
ISA-tab 13

Forward mapping

Of the 161 GHGA properties, 65 were excluded from mapping, leaving 96 for analysis. Property matches were: FAIR Genomes (50), EGA (42), ISA-tab (36), and EGA Submission API (36). Lexical matches were more frequent with the EGA model (EGA: 19, EGA-API: 17) compared to FAIR Genomes or ISA-tab (10 each). Statistical tests showed no significant association between model type and mappability (chi-square $p = 0.6409$) or GHGA class and mappability (Kruskal-Wallis $p = 0.3868$). Median mappability rates were ISA-tab, FAIR Genomes, and EGA (0.5), and EGA API (0.29). Eight GHGA classes had at least 50% mean property coverage, including *Study* (87.5%) and *Analysis* (75%). The lowest coverage occurred in *Experiment Method* (32.5%), *Research Data File* (25%), and *Publication* (17.86%). Detailed crosswalk outcomes are shown in Supplementary Figures S1 and S2.

EGA API

As shown in Fig. S1a and b, the GHGA classes *Analysis*, *Dataset*, and *Study* achieved 100% coverage in the EGA Submission API model, followed by *Individual* (60%) and *Sample* (43.7%). The lowest coverages were observed for *Experiment Method* (40%), *Analysis Method* and *Experiment* (33.33% each), *Research Data File* (25%), and *Publication* (14.29%). *Data Access Committee* and *Data Access Policy* had no corresponding properties in the EGA Submission model, nor did the supporting files or *Process Data File*.

All seven EGA Submission model classes are represented by properties in the GHGA model (Fig. S1a). The fewest connections being identified between *Research Data File* and *Run Request* (one) while *Experiment Method* and *Sample* each had seven connections to *Experiment Request* and *Sample Request*, respectively. *Sample Request* was the most frequently linked EGA class, with 10 connections – three from *Individual* and seven from *Sample classes of GHGA*. Despite having the most links, *Experiment Method* also had the highest number of unmappable properties (12), followed by *Sample* (nine), *Publication* and *Data Access Policy* (six each), and *Analysis Method* (five).

Most GHGA classes mapped to a single corresponding EGA Submission class. However, *Experiment Method* properties were split between *Analysis Request* (5%) and *Experiment Request* (35%), while *Study* properties split between *Study Request* (75%) and *Submission Request* (25%) (Fig. S1b).

Only one property recorded mappings to more than one property in the EGA API model. The *Experiment Method 'library type'* is mappable to both EGA API's *Experiment Request 'library strategy'* and *'library source'*.

FAIR Genomes

The crosswalk analysis between GHGA and the FAIR Genomes metadata model (Fig. S1c and d) showed full coverage for *Data Access Committee*, *Individual* and *Study* (100%), followed by *Data Access Policy* (83.3%), *Sample* (78.75%), and *Analysis Method* (60%). The lowest connection percentages were achieved for *Experiment* (33.33%), *Experiment Method* (30%) and *Research Data File* (25%). *Dataset* and *Publication* had no mapped properties.

Several GHGA classes - all five file types and *Experiment* - had only one mapped property with the FAIR Genomes model (Fig. S1c). The fewest connections were identified for FAIR Genomes *Clinical* (three), while the FAIR Genomes *Analysis* class was the most targeted with 15 total connections. As already observed in the comparison with the EGA API model, the GHGA *Experiment Method* is the class with the most non-mappable properties (14), followed by *Publication* (seven) and *Sample* (five).

In comparison with the crosswalk to the EGA Submission API model, more GHGA classes were mapped to two corresponding classes in the FAIR Genomes model (Fig. S1d). These are *Data Access Committee*, which is split 50-50 with FAIR Genomes *Study* and *Personal*, *Study* (75% *Study*, 25% *Personal*), *Individual* (60% *Personal*, 40% *Clinical*) and *Experiment Method*, whose properties were covered at 15% each by FAIR Genomes *Sample Preparation* and *Sequencing*. Properties in the other GHGA model classes map to only one corresponding class in the FAIR Genomes model. These are *Data Access Policy*, which maps to *Individual Consent*, *Experiment*, which maps to *Sample Preparation*, and *Analysis*, *Analysis Method*, as well as the five file classes, all of which map to FAIR Genomes *Analysis.*

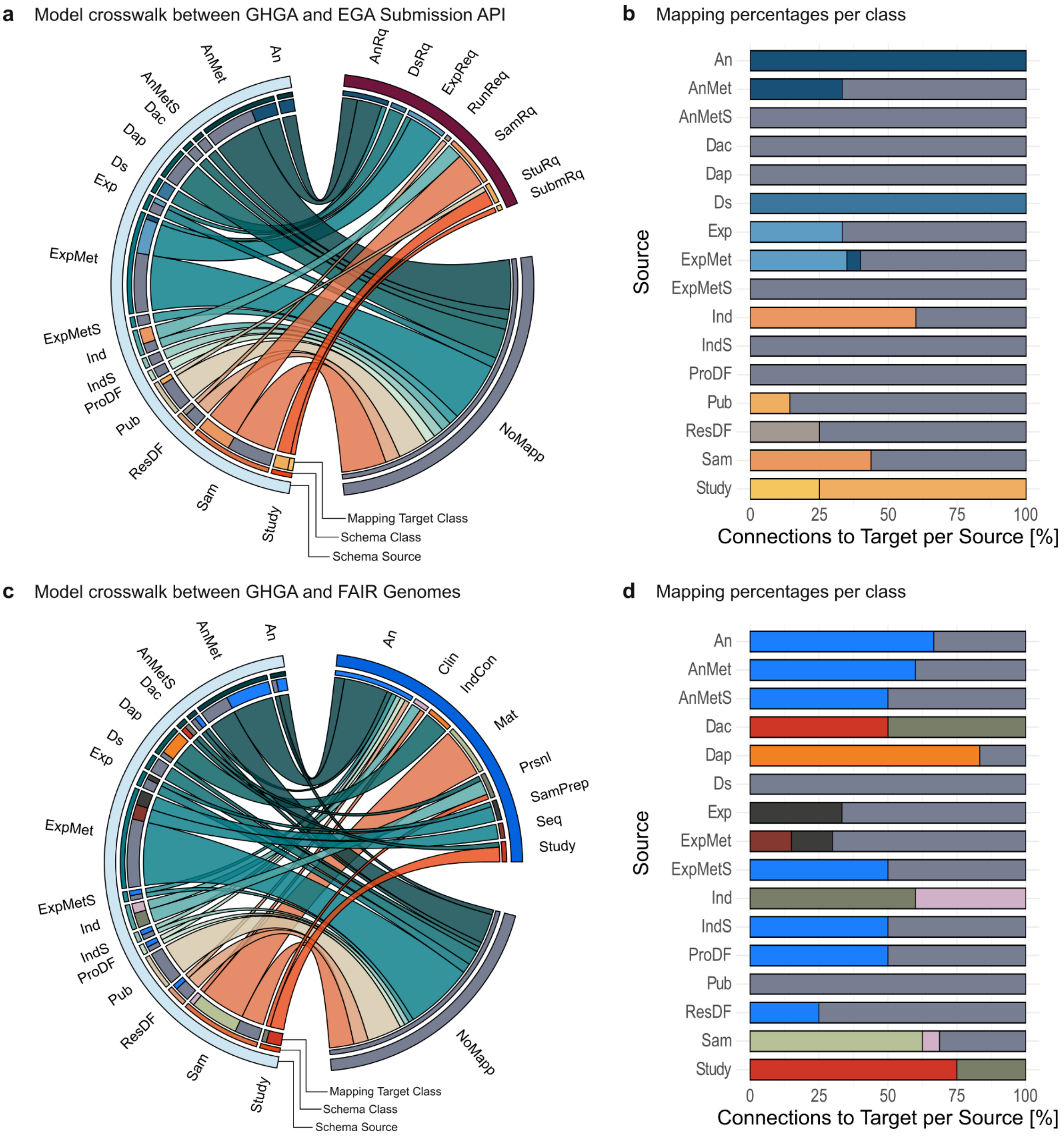

Class Abbreviations

An: Analysis; **AnMet:** Analysis Method; **AnMetS:** Analysis Method Supporting File; **AnRq:** Analysis Request; **Clin:** Clinical; **Dac:** Data Access Committee; **Dap:** Data Access Policy; **Ds:** Dataset; **DsRq:** Dataset Request; **Exp:** Experiment; **ExpMet:** Experiment Method; **ExpMetS:** Experiment Method Supporting File; **ExpReq:** Experiment Request; **Ind:** Individual; **IndCon:** Individual Consent; **IndS:** Individual Supporting File; **LeaCon:** Leaflet And Consent Form; **Mat:** Material; **NotMapp:** Not Mappable; **Prsnl:** Personal; **ProDF:** Process Data File; **Pub:** Publication; **ResDF:** Research Data File; **RunReq:** Run Request; **Sam:** Sample; **SamPrep:** Sample Preparation; **SamRq:** Sample Request; **Seq:** Sequencing; **Study:** Study; **StuRq:** Study Request; **SubmRq:** Submission Request

Schema Source

GHGA; EGA API; FAIR Genomes

Schema Class / Mapping Target Class

GHGA: Analysis; Analysis Method; Analysis Method Supporting File; Data Access Committee; Data Access Policy; Dataset; Experiment; Experiment Method; Experiment Method Supporting File; Individual; Individual Supporting File; Process Data File; Publication; Research Data File; Sample; Study

EGA API: Analysis Request; Dataset Request; Experiment Request; Run Request; Sample Request; Study Request; Submission Request

FAIR Genomes: Analysis; Clinical; Individual Consent; Leaflet and Consent Form; Material; Personal; Sample Preparation; Sequencing; Study

Not Mappable: Not Mappable

Figure S1: Forward mapping results between GHGA to EGA Submitter API and FAIR Genomes. Crosswalk circos plots (a and c) depict mapping on property level between models. Outer circles show schema sources (GHGA: light blue, EGA API: dark red, FAIR Genomes: royal blue), middle circles indicate schema classes and inner circles the mapping target class per property. Bands between classes indicate the sum of connected properties between those classes. Mapping percentages per class (b and d) zoom into the circos plot inner circles (representing the mapping target classes) and depict the connections as percentage.

EGA

The crosswalk between GHGA and the EGA model (Fig. S2a and b) yielded results similar to those from the GHGA and the EGA Submission API comparison. GHGA's *Analysis* and *Dataset* classes achieved 100% mapping, with full coverage also for the *Experiment* class. *Data Access Policy* (83.33%), *Study* (75%), *Sample* (62.5%), and *Individual* (60%) mapped above 50%. For *Data Access Policy* and *Experiment Method Supporting File*, 50% of properties mapped to equivalents in EGA. The lowest mappings were for *Experiment Method* (35%) and *Analysis Method* (20%). No mappings were found for *Analysis Method Supporting File*, *Individual Supporting File*, *Process Data File*, *Research Data File*, and *Publication*.

As depicted in Fig. S2a, the EGA *Sample* class was most frequently targeted, with 10 links from GHGA *Sample,* followed by *Assay* with nine (GHGA *Experiment*: five, *Experiment Method*: three, *Experiment Method Supporting File*: one). *Experiment Method* had the most non-mappable properties (13), followed by *Analysis Method* (12), *Publication* (seven), *Sample* (six), and *Research Data File* (four).

Class-level mappings were mostly one-to-one. The only exception was GHGA *Experiment Method*, which split between EGA *Experiment* (10%) and *Assay* (25%) (Fig. S2b). All other GHGA classes mapped directly to single EGA classes: *Analysis* and *Analysis Method* to *Analysis*, *Data Access Committee* and *Data Access Policy* to their equivalents, *Dataset* to *Dataset*, *Experiment* and its *Supporting File* to *Experiment*, and *Individual*, *Sample*, and *Study* to their lexical matches.

ISA-tab

The crosswalk analysis between GHGA and ISA-tab (Fig. S2c and 3d), produced different results than prior analyses. Five GHGA classes, including all three auxiliary file types, achieved full coverage with the ISA-tab model. These include the supporting files, *Process Data File* and *Data Access Committee*. *Study* (75%) and *Publication* (57.14%) had over 50% mapping. *Research Data File* and *Analysis Method* mapped at exactly 50%, while *Analysis*,

Experiment (33.33% each), *Experiment Method* (25%), and *Sample* (12.5%) were below 50%. No mappable properties were found for *Data Access Policy*, *Dataset*, and *Individual*.

Some GHGA classes had only one or two properties mapped to the ISA-tab model (Fig. S2c) such as the three supplementary files, *Process Data File* and *Sample* (two connections each), and *Analysis* and *Experiment* (one connection each). Four ISA-tab classes - *Organization*, *Protocol*, *Sample*, and *Source* - were targeted only once. ISA-tab *Protocol* was the most targeted with 11 links (eight from GHGA *Analysis Method*, three from *Experiment Method*), followed by ISA-tab *Data* (six), and *Investigation* and *Publication* (four each). As observed in all previous comparisons, GHGA *Experiment Method* again had the most non-mappable properties (15), followed by *Sample* (14), *Analysis Method* (eight), *Data Access Policy* (six), and *Individual* (five).

Of the 13 GHGA classes with mappings, six mapped fully to a single ISA-tab class: *Analysis* to *Process*, *Analysis Method* to *Protocol*, *Data Access Committee* to *Person*, *Experiment* to *Assay*, *Publication* to *Publication*, and *Research Data File* to *Data*. The three supporting file types were split evenly between *Investigation* and *Data*. *Experiment Method* properties were split between *Protocol* (15%) and *Assay* (10%), and *Sample* mapped partially to both *Sample* and *Source* (6.25% each). *Study* mapped to ISA-tab *Study* (50%) and *Person* (25%) (Fig. S2d).

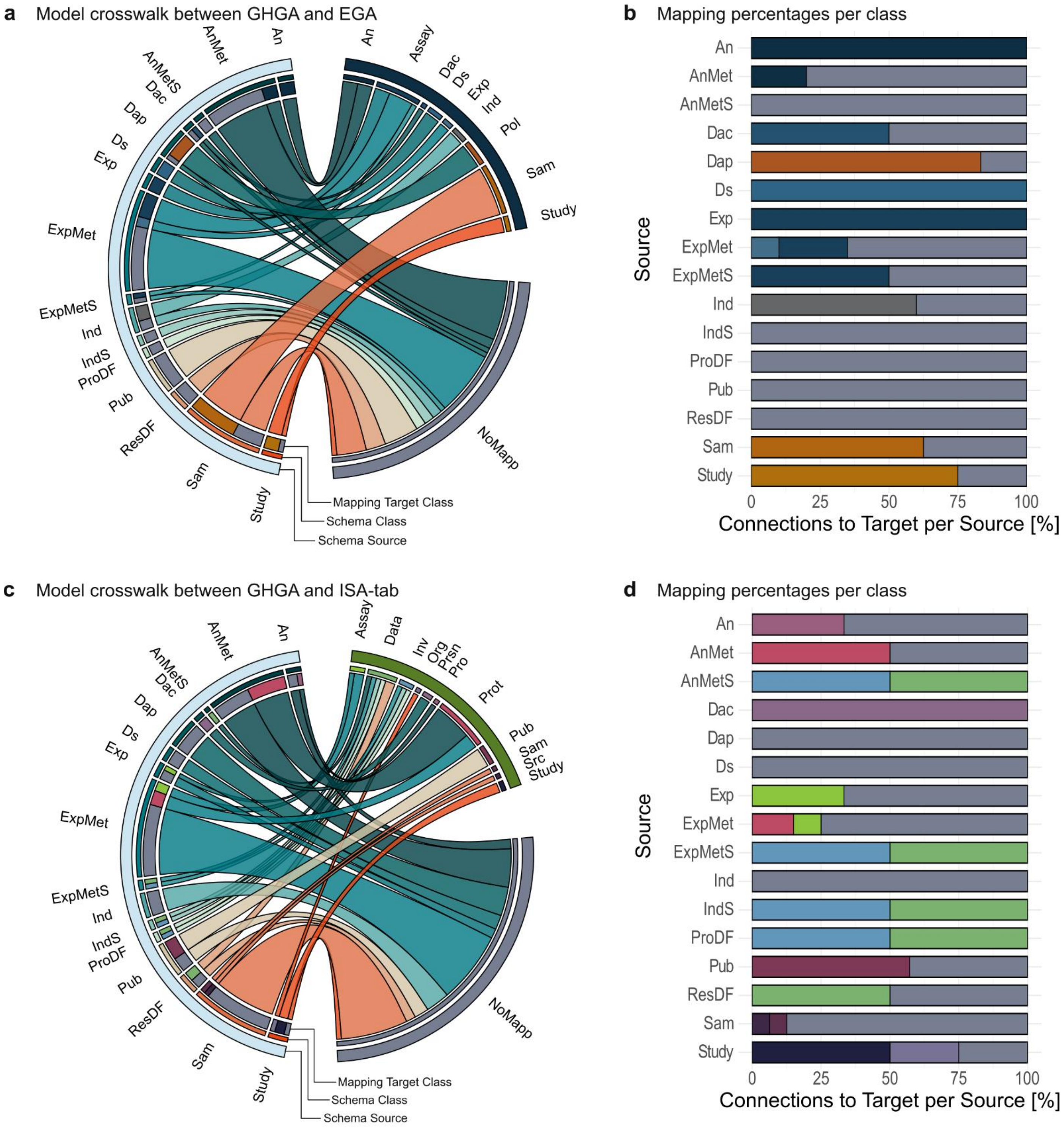

Class Abbreviations

An: Analysis; **AnMet:** Analysis Method; **AnMetS:** Analysis Method Supporting File; **Assay:** Assay; **Dac:** Dac / Data Access Committee; **Data:** Data; **Dap:** Data Access Policy; **Ds:** Dataset; **Exp:** Experiment; **ExpMet:** Experiment Method; **ExpMetS:** Experiment Method Supporting File; **Ind:** Individual; **IndS:** Individual Supporting File; **Inv:** Investigation; **Mat:** Material; **NotMapp:** Not Mappable; **Org:** Organization; **Prsn:** Person; **Pol:** Policy; **Pro:** Process; **ProDF:** Process Data File; **Prot:** Protocol; **Pub:** Publication; **ResDF:** Research Data File; **Sam:** Sample; **SamPrep:** Sample Preparation; **Seq:** Sequencing; **Src:** Source; **Study:** Study

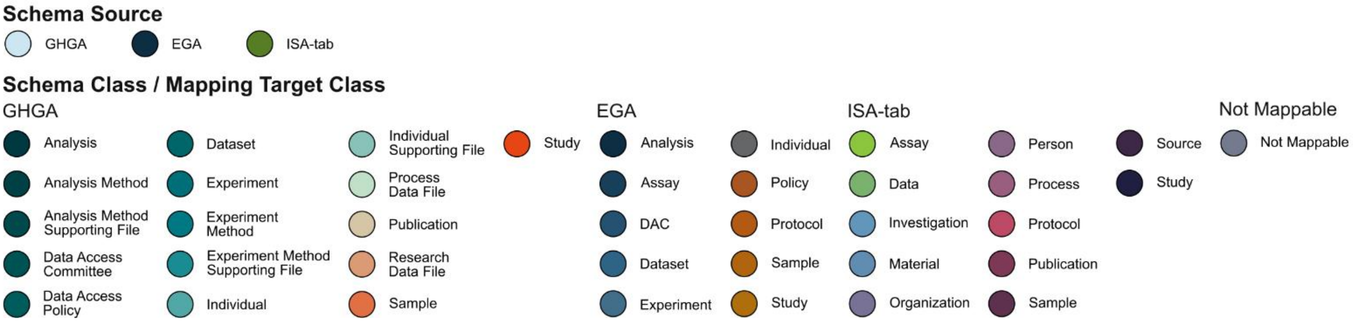

Figure S2: Forward mapping results between GHGA to EGA and ISA-tab. Crosswalk circos plots (a and c) depict mapping on property level between models. Outer circles show schema sources (GHGA: light blue, EGA: dark blue, ISA-tab: green), middle circles indicate schema classes and inner circles the mapping target class per property. Bands between classes indicate the sum of connected properties between those classes. Mapping percentages per class (b and d) zoom into the circos plot inner circles (representing the mapping target classes) and depict the connections as percentages.

Backward mapping

Properties excluded before mapping were: 52 of 91 from EGA Submission, 20 of 112 from FAIR Genomes, 56 of 117 from EGA, and 144 of 184 from ISA-tab. The total matches identified were EGA API (48), EGA (35), FAIR Genomes (33), and ISA-tab (25). Counts of unmatched properties were two (EGA API), 13 (EGA), 15 (ISA-tab), and 59 (FAIR Genomes). Exact matches dominated: EGA API (25), EGA (42), FAIR Genomes (23), and ISA-tab (21); narrow matches occurred only once in EGA API, EGA, and FAIR Genomes. EGA displayed the most total (48) and lexical (20) exact matches, while EGA API had the fewest unmatched properties and 15 lexical exact matches. FAIR Genomes and ISA-tab each had 10 lexical exact matches. A chi-square test confirmed a significant relationship between model and mappability ($\chi^2 = 23.362$, df = 3, $p = 3.393 \times 10^{-5}$).

GHGA classes common to all comparisons included *Analysis Method*, *Experiment Method*, *Sample*, and *Study*. *Individual*, *Data Access Committee*, and *Research Data File* appeared in three models; *Data Access Policy*, *Publication*, and *Dataset* in two; *Analysis*, *Experiment*, and *Process Data File* in one. Median mappability for common classes was highest for ISA-tab and EGA-API (1.0), EGA (0.83), and FAIR Genomes (0.46). A Kruskal-Wallis test detected significant differences in mappability ($\chi^2 = 12.536$, df = 3, $p = 0.005755$). EGA Submission API showed the highest mean coverage (97.14%), followed by EGA (78.93%), ISA-tab (70.69%), and FAIR Genomes (40.16%). Several classes reached 100% coverage: *Dataset Request*, *Run Request*, *Sample Request, Analysis Request*, *Study Request*, and *Submission Request* (EGA API); *Analysis*, *Policy*, *Protocol*, and *Study* (EGA); and *Assay*, *Data*, *Organization*, *Protocol*, *Sample*, and *Source* (ISA-tab). FAIR Genomes *Leaflet and Consent Form*, and ISA-tab *Material* had no GHGA equivalent. No FAIR Genomes class exceeded 50% alignment. Full mapping details and figures are available in Supplementary Figures S3 and S4.

EGA API

As shown in Fig. S3a and b, six out of seven classes achieved 100% coverage with the GHGA metadata model. *Experiment Request* reached 80%. The crosswalk revealed links between all seven EGA classes and eight of GHGA's 16 classes: *Analysis Method*, *Dataset*, *Experiment Method*, *Individual*, *Publication*, *Research Data File*, *Sample*, and *Study*. No mappings were found for *Analysis*, *Data Access Committee*, *Data Access Policy*, *Experiment*, *Process Data File*, or the three auxiliary files.

Fewest links occurred between EGA API *Study Request* and GHGA *Publication*, as well as between EGA API *Run Request* and GHGA *Research Data File* (one property each), followed by EGA API *Sample* and GHGA *Individual* (two). The strongest mappings were between *Analysis Request* and the methodological classes (*Analysis Method*: four properties, *Experiment Method*: five properties), *Experiment Request* and *Experiment Method* (eight), and *Sample* and *Sample* (seven). The EGA *Experiment* class had the most connections overall (12). Only two of 37 EGA properties could not be mapped: 'paired nominal length' and 'paired nominal sdev' (*Experiment Request*).

Properties in the *Dataset Request*, *Run Request* and *Submission Request* classes could be mapped to one respective corresponding class in the GHGA metadata model, while fields in the *Analysis Request*, *Sample Request* and *Study Request* classes are split between two GHGA classes each (Fig. S3a and b). *Analysis Request* mapped to properties in the GHGA classes *Analysis Method* (44.44%) and *Experiment Method* (55.56%). Properties in the *Sample Request* were split between GHGA *Sample* (80%) and *Individual* (20%), while fields in the *Study Request* were divided between *Study* (75%) and *Publication* (25%) (Fig. S3b).

Three EGA *Analysis Request* properties – 'title', 'description', and 'analysis type' – had multiple equivalents in GHGA (e.g., 'title' or 'name', 'description', 'type') across *Analysis* and *Analysis Method* classes.

FAIR Genomes

The crosswalk analysis between FAIR Genomes and GHGA shown in Figure S3c and d revealed a maximum mapping coverage of 50% across all FAIR Genomes model classes. *Personal*, *Sequencing* and *Study* had the highest mapping percentage, followed by *Material* with 46.15% coverage, *Analysis* (44.44%) and *Individual Consent* (33.33%). *Sample Preparation* (28.57%) and *Clinical* (18.75%) had the lowest coverage, while *Leaflet and Consent Form* were the only classes that had no equivalent property in the GHGA model.

All FAIR Genomes classes except *Leaflet and Consent Form* recorded at least two connecting properties with the GHGA model (Fig. S3c). FAIR Genomes classes *Personal* and *Material* had the highest number of connections with six properties, followed by *Sequencing* (five), *Study* and *Analysis* (four each). *Individual Consent* and *Clinical* recorded three connections each, and *Sample Preparation* two.

Similar to the forward mapping between GHGA and FAIR Genomes, classes in the two models were more heterogeneous, resulting in class mapping splits between two or more classes (Fig. S3d). While *Individual Consent*, *Sample Preparation* and *Sequencing* only map to one GHGA class (*Individual Consent*: *Data Access Policy*, *Sample Preparation* and *Sequencing*: *Experimental Method*), all other classes' properties split between at least two GHGA model classes. Properties in the FAIR Genomes *Analysis* class map to GHGA *Process Data File* (11.11%) and *Analysis Method* (33.33%), whereas FAIR Genomes *Study* splits between GHGA *Study* (37.5%) and *Data Access Committee* (12.5%). A similar split can be observed between FAIR Genomes *Clinical* and *Personal*, both of which map to GHGA *Sample* (*Clinical*: 6.25%, *Personal*: 8.33%) and *Individual* (*Clinical*: 12.5%, *Personal*: 33.33%). However, FAIR Genomes *Personal* additionally maps to GHGA *Data Access Committee* (8.33%), making it the only property with a three-way class split. Lastly, FAIR Genomes *Material* maps to GHGA *Sample* (38.46%) and *Experiment Method* (7.69%).

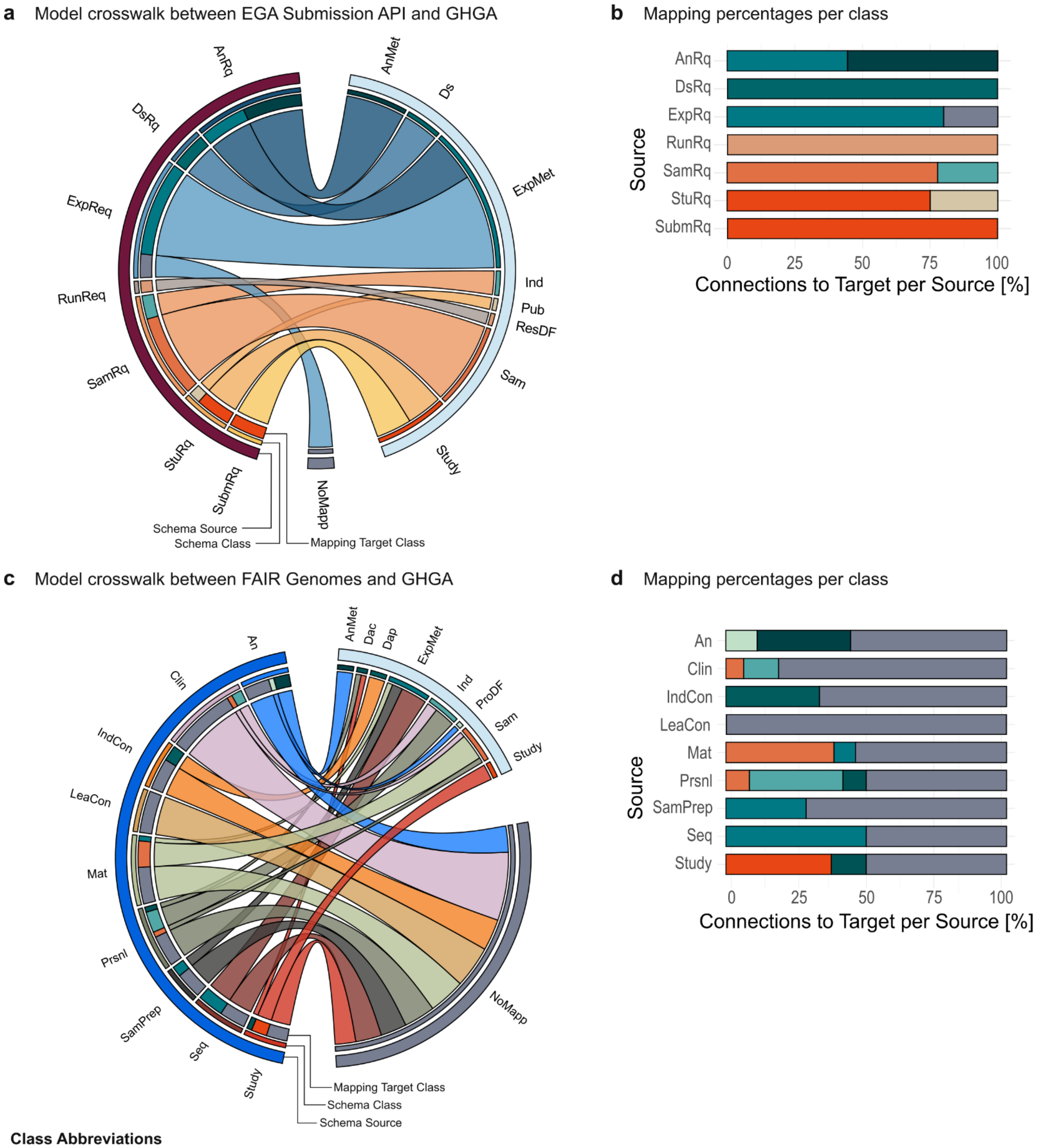

Class Abbreviations

An: Analysis; **AnMet:** Analysis Method; **AnMetS:** Analysis Method Supporting File; **AnRq:** Analysis Request; **Clin:** Clinical; **Dac:** Data Access Committee; **Dap:** Data Access Policy; **Ds:** Dataset; **DsRq:** Dataset Request; **Exp:** Experiment; **ExpMet:** Experiment Method; **ExpMetS:** Experiment Method Supporting File; **ExpReq:** Experiment Request; **Ind:** Individual; **IndCon:** Individual Consent; **IndS:** Individual Supporting File; **LeaCon:** Leaflet And Consent Form; **Mat:** Material; **NotMapp:** Not Mappable; **Prsnl:** Personal; **ProDF:** Process Data File; **Pub:** Publication; **ResDF:** Research Data File; **RunReq:** Run Request; **Sam:** Sample; **SamPrep:** Sample Preparation; **SamRq:** Sample Request; **Seq:** Sequencing; **Study:** Study; **StuRq:** Study Request; **SubmRq:** Submission Request

Schema Source

GHGA · EGA API · FAIR Genomes

Schema Class / Mapping Target Class

GHGA: Analysis; Analysis Method; Analysis Method Supporting File; Data Access Committee; Data Access Policy; Dataset; Experiment; Experiment Method; Experiment Method Supporting File; Individual; Individual Supporting File; Process Data File; Publication; Research Data File; Sample; Study

EGA API: Analysis Request; Dataset Request; Experiment Request; Run Request; Sample Request; Study Request; Submission Request

FAIR Genomes: Analysis; Clinical; Individual Consent; Leaflet and Consent Form; Material; Personal; Sample Preparation; Sequencing; Study

Not Mappable: Not Mappable

Figure S3: Backward mapping results between EGA Submitter API to GHGA and FAIR Genomes to GHGA. Crosswalk circos plots (a and c) depict mapping on property level between models. Outer circles show schema sources (GHGA: light blue, EGA API: dark red, FAIR Genomes: royal blue), middle circles indicate schema classes and inner circles the mapping target class per property. Bands between classes indicate the sum of connected properties between those classes. Mapping percentages per class (b and d) zoom into the circos plot inner circles (representing the mapping target classes) and depict the connections as percentage.

EGA

The crosswalk analysis between EGA and GHGA (Fig. S4a and b) mirrors the results seen in the mapping between GHGA and EGA Submission API. Five EGA classes achieved 100% mapping (*Analysis*, *Experiment*, *Policy*, *Protocol*, *Study*), while only the Data *Access Committee* had less than 50% overlap (33.33%). *Assay* and *Individual* reached 57.15%, *Sample* 66.67%, and *Dataset* 75%. All EGA classes had at least one mappable property.

As highlighted in Fig. S4a, the mappings were found for 11 of 16 GHGA classes; no mappings were recorded for *Analysis*, *Publication*, and the three supplementary files. The most connections originated from EGA *Sample* (eight), followed by *Protocol* and *Experiment* (seven each), then *Analysis* (six), and *Study*, *Policy*, *Individual*, and *Assay* (four each). *The Dataset* had three, and the Data *Access Committee* had only one. On the GHGA side, *Experiment Method* was the most targeted class (10 connections), followed by *Analysis Method* and *Sample* (eight each), and *Study* (five). *The Data Access Committee*, *Process Data File*, *Experiment*, and *Research Data File* had the fewest (1–2).

Class-level mappings were uniform for six out of the 10 EGA classes (*Data Access Committee*, *Dataset*, *Individual*, *Policy*, *Sample*, *Study*), all of which mapped to lexically matching classes in the GHGA model (*Data Access Committee*, *Dataset*, *Individual*, *Data Access Policy*, *Sample*, *Study*) (Fig. S4b). The remaining EGA classes mapped to multiple GHGA classes. *Assay* split between *Experiment* and *Experiment Method* (28.57% each), while *Experiment* mapped mostly to *Experiment Method* (66.67%) and partially to *Research Data File* (11.11%). *Protocol* properties aligned with *Analysis Method* (71.43%), and partially with *Experiment Method* and *Study* (14.29% each). *Analysis* linked to four GHGA classes: *Analysis Method* (50%), and *Process Data File*, *Research Data File*, and *Experiment Method* (16.67% each).

Three EGA properties had multiple GHGA equivalents: *Policy 'duoCodes'* corresponds to both *Data Access Policy 'data use permission term'* and *'data use modifier term', Protocol 'software'*

to both *Analysis Method ‘software’* or *‘workflow name’*. *Sample ‘sampleTypes’* to GHGA *Sample ‘type’* or *‘biospecimen type’*.

ISA-tab

The crosswalk analysis between ISA-tab and GHGA achieved connections to eight of the 16 GHGA model classes (Fig. S4c and d). No mappings were found for GHGA *Data Access Policy*, *Dataset*, *Experiment*, *Individual*, *Process Data File*, and the three supplementary file classes. Six of the 12 ISA-tab classes achieved 100% coverage: *Assay*, *Data*, *Organization*, *Protocol*, *Sample*, and *Source*. *Investigation*, *Publication*, and *Study* reached over 50% mappability, while *Person* and *Process* were below 50%. *Material* had no mappings.

Several ISA-tab model classes had only one or two connections to the GHGA model; *Source*, *Sample*, *Process*, *Organization* (one each) and *Study*, *Person* and *Data* (two each) as seen in (Fig. S4c). *Assay* and *Investigation* had three each, *Publication* had four, and *Protocol* five. GHGA *Experiment Method* was the most targeted (six connections), followed by *Study* (five) and *Publication* (four). *Protocol* and *Assay* linked to *Experiment Method*, while *Study* was targeted by *Study*, *Investigation* (two each), and *Organization* (one).

Out of 11 ISA-tab classes with GHGA mappings, nine had one-to-one mappings (Fig. S4d). *Sample* and *Source mapped to* GHGA *Sample* (100%), *Organization* and *Study* to *GHGA Study,* and *Assay, Data, Person, Process,* and *Publication* to their respective counterparts. *Assay* mapped to *Experiment Method, Data* to *Research Data File, Person* to *Data Access Committee* (25%), *Process* to *Analysis* (33.33%), and *Publication* to *Publication* (80%). *Investigation* mapped to *Study* (40%) and *Research Data File* (20%); *Protocol* to *Analysis Method* (40%) and *Experiment Method* (60%).

Four ISA-tab properties had multiple GHGA equivalents. *Investigation*'s *‘filename’* mapped to all five GHGA file name fields, while *Protocol*’s ‘name’, ‘protocol type’, and ‘description’ aligned with GHGA *Analysis Method* or *Experiment Method* equivalents.

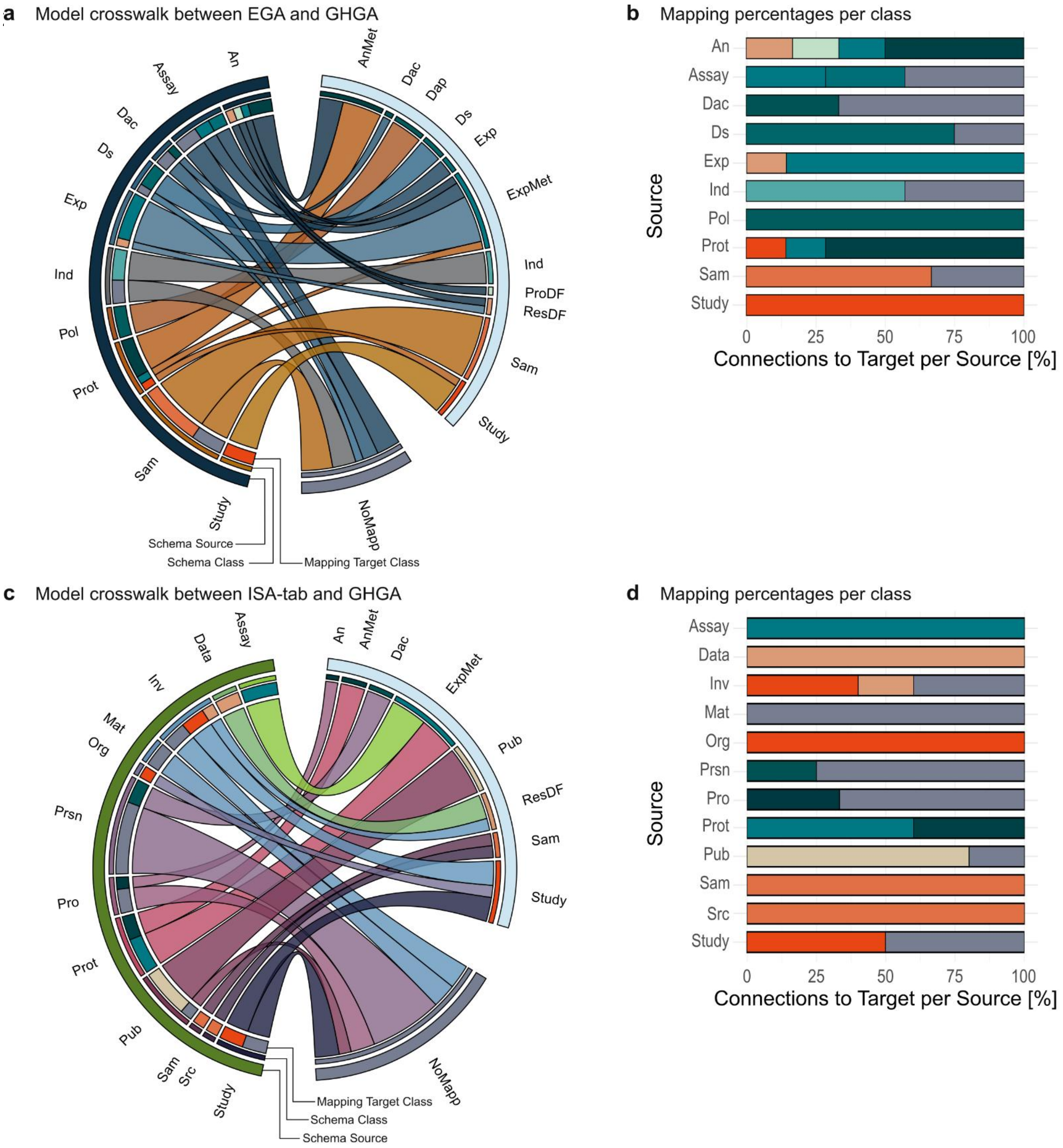

Class Abbreviations

An: Analysis; **AnMet:** Analysis Method; **AnMetS:** Analysis Method Supporting File; **Assay:** Assay; **Dac:** Dac / Data Access Committee; **Data:** Data; **Dap:** Data Access Policy; **Ds:** Dataset; **Exp:** Experiment; **ExpMet:** Experiment Method; **ExpMetS:** Experiment Method Supporting File; **Ind:** Individual; **IndS:** Individual Supporting File; **Inv:** Investigation; **Mat:** Material; **NotMapp:** Not Mappable; **Org:** Organization; **Prsn:** Person; **Pol:** Policy; **Pro:** Process; **ProDF:** Process Data File; **Prot:** Protocol; **Pub:** Publication; **ResDF:** Research Data File; **Sam:** Sample; **SamPrep:** Sample Preparation; **Seq:** Sequencing; **Src:** Source; **Study:** Study

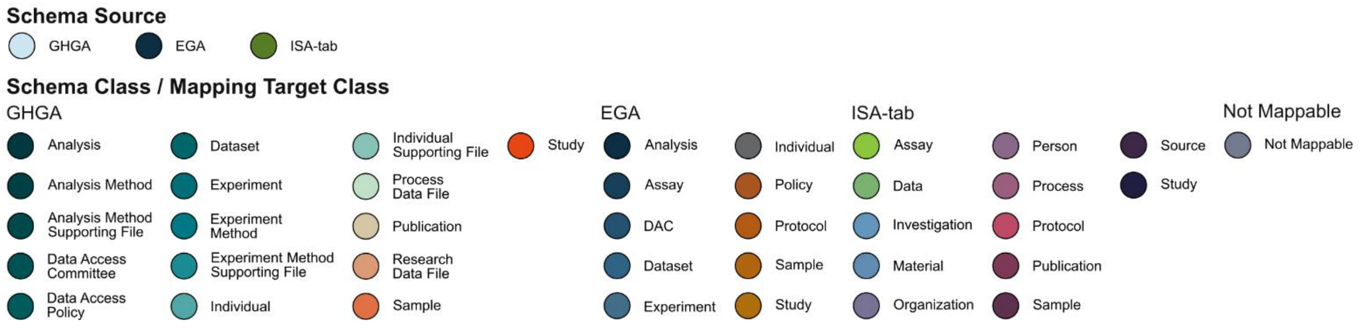

Figure S4: Backward mapping results between EGA to GHGA and ISA-tab to GHGA. Crosswalk circos plots (a and c) depict mapping on property level between models. Outer circles show schema sources (GHGA: light blue, EGA: dark blue, ISA-tab: green), middle circles indicate schema classes and inner circles the mapping target class per property. Bands between classes indicate the sum of connected properties between those classes. Mapping percentages per class (b and d) zoom into the circos plot inner circles (representing the mapping target classes) and depict the connections as percentage.
